# The Malaria Burden: A South African Perspective

**DOI:** 10.1155/2024/6619010

**Published:** 2024-02-23

**Authors:** Marissa Balmith, Charlise Basson, Sarel J. Brand

**Affiliations:** ^1^Department of Pharmacology, School of Medicine, University of Pretoria, Pretoria, South Africa; ^2^Department of Physiology, School of Medicine, University of Pretoria, Pretoria, South Africa; ^3^Center of Excellence for Pharmaceutical Sciences, Department of Pharmacology, North-West University, Potchefstroom, South Africa

## Abstract

Malaria is a deadly disease caused by protozoan pathogens of the *Plasmodium* parasite. Transmission to humans occurs through the bite of an infected female *Anopheles* mosquito. According to the World Health Organization (WHO), an estimated 247 million cases of malaria were recorded worldwide in 2021, with approximately 619 000 malaria deaths. The initial signs of malaria can be mild and challenging to diagnose due to the signs and symptoms being similar to those of other illnesses. The malaria burden remains largely concentrated in the WHO sub-Saharan African region and has been recognised as a significant contributor to morbidity and mortality. This review aims to contribute to the existing knowledge on malaria in South Africa, a region within sub-Saharan Africa, focusing on the epidemiology and life cycle of the malaria parasite as well as diagnostic approaches for detecting malaria. In addition, nonpharmacological and pharmacological interventions for treating and preventing malaria infections will also be discussed herein. While there has been a significant reduction in the global burden of this disease, malaria remains a public health issue in South Africa. As such, the implementation of effective preventative measures and strategies, early diagnosis, and appropriate treatment regimens are crucial to reducing the malaria burden in South Africa.

## 1. Introduction

Malaria is a life-threatening infectious disease and is considered one of the leading contributors to the ongoing global health crisis. Malaria, which is caused by the *Plasmodium* parasite, is a mosquito-borne disease that spreads from person to person through the bite of an infected female *Anopheles* mosquito [1]. In 2021, approximately half of the global population was at risk for malaria, with the World Health Organization (WHO) African region accounting for 95% of the global malaria burden [2]. In addition, malaria was responsible for approximately 80% of deaths in children under the age of five on the WHO African continent [2]. It is important to note that while there are several different species of the *Plasmodium* parasite, only five are known to cause malaria in humans, namely, *Plasmodium falciparum* (*P. falciparum*), *Plasmodium vivax* (*P. vivax*), *Plasmodium malariae* (*P. malariae*), *Plasmodium ovale* (*P. ovale*), and *Plasmodium knowlesi* (*P. knowlesi*) [3]. Of these, *P. falciparum* is the most common in humans [2], with African countries, including South Africa, bearing the burden of *P. falciparum* infections [4], due to factors such as high transmission rates and socioeconomic challenges [5] that limit their access to healthcare services [3, 6]. Of note, *P. falciparum* is widely considered to be the most virulent and potentially fatal if not promptly diagnosed and treated [7–9]. As such, this review will focus on the malaria burden in South Africa, a country located in the southernmost part of the African continent in sub-Saharan Africa, which has been stricken by at least two major outbreaks in the last two decades, resulting in numerous people succumbing to the disease [10]. By providing an in-depth understanding of the malaria burden in South Africa, this review aims to contribute to the ongoing global efforts in the fight against this devastating disease.

## 2. Epidemiology of Malaria in South Africa

**Figure 1:**
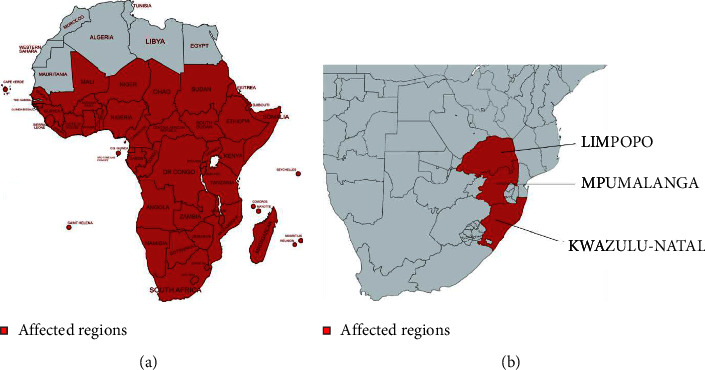
A map of regions affected by malaria transmission in (a) sub-Saharan Africa and (b) provinces affected by malaria in South Africa, including Limpopo, Mpumalanga, and KwaZulu-Natal [12, 13].

The transmission of malaria is closely linked to specific ecological conditions [11]. In South Africa, malaria infections are almost exclusively caused by *P. falciparum* and remain endemic to the northeastern parts of the country in certain South African provinces, including Limpopo, KwaZulu-Natal, and Mpumalanga (Figure 1) [12], especially during the humid and rainy months of summer [12, 14].

## 3. Malaria Statistics in South Africa

Over the past decades, South Africa has shown consistent improvement in reducing both the morbidity and mortality rates associated with malaria [15]. For many years, annual malaria cases in South Africa were maintained below 10,000 due to vector control and case management efforts, with approximately 8750 reported cases in 1995 [12]. However, beginning in 1996, the effectiveness of insecticides and treatments decreased, leading to a sharp increase in malaria cases and deaths that peaked in 2000 [12]. During this period, malaria cases rose by 67% in 1996 and reached over 60 000 in 2000, resulting in more than 400 deaths [12]. The decline in efficacy of sulphadoxine-pyrimethamine might have contributed to the significant increase in mortality [12].

In 2000, when the first-line malaria drug sulphadoxine-pyrimethamine failed in South Africa, they reintroduced dichlorodiphenyltrichloroethane (DDT) for traditional structures while maintaining pyrethroids for modernised housing, using a mosaic strategy for resistance management [16, 17]. Artemisinin-containing combination treatment (ACT) was also introduced for malaria treatment [17, 18]. Despite global pressure against insecticide use, South Africa decided to bring back DDT to control the malaria epidemic [17, 19]. Following the adoption of regional malaria control strategies in South Africa, Swaziland, and Mozambique, the implementation of ACTs and the introduction of an effective insecticide, national case numbers decreased to 26 506 in 2001 [12]. In 2007, South Africa reported fewer than 6000 malaria cases and subsequently started internal discussions on malaria elimination based on WHO recommendations [20, 21]. These statistics continued to fall to below 10 000 by 2011 [12]. In 2012, South Africa formally adopted an elimination strategy aiming to stop local malaria transmission within the country's borders by 2018 [20, 22]. South Africa has achieved an 87% reduction in malaria cases, with a decline from 64,622 cases in 2000 [17] to 8,126 cases in 2020 [15]. Furthermore, the number of malaria-related deaths has decreased by 91% (459 deaths in 2000 to 38 deaths in 2020) [15].

## 4. Factors Influencing the Transmission of Malaria in South Africa

### 4.1. Vector

The predominant vector for malaria in South Africa is the *Anopheles* mosquito [23], which uses stagnant water sources like ponds, puddles, and irrigated fields to create the ideal breeding ground [24, 25]. Given the variations in breeding preferences among *Anopheles* mosquitoes, their prevalence and distribution are influenced by numerous factors such as rainfall patterns, temperature, soil characteristics, vegetation cover [26], and human activities (e.g., deforestation and migration) [27]. Areas with higher vector populations are at a greater risk of malaria transmission, especially when combined with other factors such as the presence of infected individuals and inadequate protective measures [24]. While infection with *P. falciparum* continues to be the primary cause of malaria infections in South Africa, effective control measures have aided in successfully suppressing malaria infection rates until the early 1980s [12]. As a result, South Africa established a monitoring system aimed at assessing the *in vitro* effectiveness of first-line treatments and gaining insights into the impact of drug resistance on the ever-changing malaria trends [12]. In South Africa, efforts to control malaria focus on targeting these vectors with the use of measures like (i) indoor residual spraying (IRS), which involves applying an insecticide to the walls and surfaces of a house where the insecticide remains active for several months, effectively eliminating mosquitoes that come into contact with the treated areas [28], (ii) the distribution of insecticide-treated bed nets (ITNs) which creates a protective barrier against mosquitoes and aids in repelling and eliminating mosquitoes [29], and (iii) larviciding by destroying larval habitats [30]. These measures aim to reduce vector populations, limit human-mosquito contact, and disrupt the cycle of transmission.

### 4.2. Environmental Factors

Climatic factors heavily influence the distribution of malaria, such as high temperatures, humidity, and rainfall [31]. Malaria occurs predominantly in tropical and subtropical areas where the *Anopheles* mosquito can survive and reproduce, allowing the malaria parasite to complete its life cycle in the mosquito [32]. Elevated temperatures have been known to lead to the production of smaller and fecund mosquitoes [33]. As temperatures rises, the maturation period for mosquitoes decreases, while their feeding frequency increases [24]. Thus, temperature is crucial, as *P. falciparum* is unable to complete its growth cycle in the *Anopheles* mosquito at cooler temperatures below 20°C and as a result, cannot be transmitted [34]. Transmission is favourable in warmer regions, with the highest transmission found in sub-Saharan Africa (Figure 1) [11, 34]. In South Africa, malaria transmission increases around the month of October, reaching a peak during the months of January and February, followed by a decline in May [35]. Notably, South Africa's climate plays a significant role in shaping the malaria statistics in the country.

### 4.3. Human Behaviour

Human behaviour is also known to play a significant role in the transmission of malaria. Individuals living in malaria-endemic regions are often more exposed to mosquito bites due to various factors, including sleeping outdoors, going outdoors at night [36], inadequate utilisation of mosquito nets, or limited access to protective or preventative measures [37]. Furthermore, population movement and travel, such as migration, tourism, and labour migration to malaria-endemic areas, can introduce or spread malaria parasites to previously unaffected regions [38, 39]. It is also important to consider the impact of human behaviour on the transmission of malaria in South Africa. During the peak transmission period, human behaviour and practices, such as travel [40, 41], outdoor activities (such as farming) [42], and the use of protective measures [43], play a crucial role in the spread of malaria [40]. In addition, compliance with protective measures, such as the use of insecticide-treated nets and antimalarial medication, can also influence the dynamics of malaria transmission [44]. In addition, cultural practices and community perceptions in South Africa are also known to influence the spread of malaria [45]. As such, it is important to consider targeted interventions and preventive measures to help mitigate malaria transmission. Access to malaria diagnostic services in South Africa during the peak transmission period is particularly crucial for disease management. Healthcare facilities need to be well-equipped with diagnostic technologies and services. However, the South African healthcare system grapples with resource constraints, overburdened facilities, and a high burden of infectious diseases, such as human immunodeficiency virus (HIV)/acquired immunodeficiency syndrome (AIDS) and other noncommunicable diseases, placing additional pressure on an already strained healthcare system [46, 47].

### 4.4. Socioeconomic Factors and Vulnerable Populations

Socioeconomic conditions also influence malaria transmission in South Africa. These include a lack of education [5], limited access to healthcare and diagnostic services [48], inadequate housing facilities, and poverty [5]. A delay in diagnosis and treatment often leads to the progression of infection and further transmission within the community [49, 50]. Socioeconomic factors majorly influence vulnerable populations, with children under the age of 5 in the sub-Saharan Africa region accounting for two-thirds of these deaths [51]. It is estimated that 1% of children infected with *P. falciparum* will develop cerebral malaria [52]. Furthermore, it is well known that HIV increases an individual's susceptibility to malaria [53] and that South Africa is among the many countries predominantly affected by the global HIV burden [54]. Pregnant women are among the most vulnerable, and pregnancy-associated malaria may lead to severe malaria, cerebral malaria, anaemia, premature birth, abortions, low birthweight babies, congenital malaria, and a higher risk of coinfections [55]. From the authors' perspective, in a developing country like South Africa, people living in poverty or with poor access to healthcare may also be more likely to contract malaria due to factors such as inadequate housing, water, sanitation, hygiene, and limited access to effective antimalarial treatments.

### 4.5. Malaria Life Cycle

**Figure 2:**
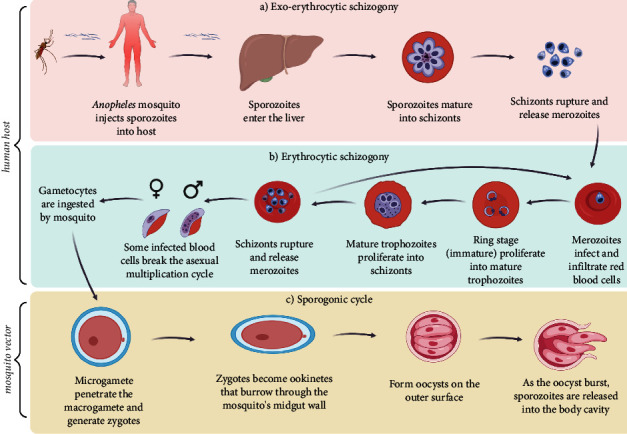
The malaria life cycle [56]. The image was created using BioRender (https://biorender.com/).

The life cycle of the malaria parasite is complex and involves two hosts, namely, human (Figures 2(a) and 2(b)) and vector (mosquito) (Figure 2(c)). A malaria-infected female *Anopheles* mosquito injects sporozoites into the human host during a blood meal. Sporozoites enter the liver and infect liver cells (hepatocytes) [6, 56]. Sporozoites then mature into schizonts and rupture, releasing merozoites [6, 56]. Following replication in the liver, in a process known as exoerythrocytic schizogony (Figure 2(a)), the parasites undergo asexual multiplication in the erythrocytes (erythrocytic schizogony) (Figure 2(b)) [6, 56]. Merozoites infect and infiltrate RBCs (erythrocytes) [6, 56] (Figure 2). The ring-stage trophozoites proliferate into mature trophozoites, which then mature into schizonts. The latter rupture and release merozoites, which infect erythrocytes [6, 56]. Some infected blood cells break the asexual multiplication cycle. Instead of replicating, the merozoites in these cells mature into gametocytes, which circulate in the bloodstream as sexual forms of the parasite [6, 56]. When a mosquito bites an infected person, the gametocytes are ingested. The parasites' multiplication in the mosquito is known as the sporogonic cycle [6, 56] (Figure 2(c)). While in the mosquito's stomach, the microgametes penetrate the macrogametes, generating zygotes [6, 56]. Zygotes become motile and elongated (ookinetes) that burrow through the mosquito's midgut wall and form oocysts on the outside surface [6, 56]. As the oocyst bursts, sporozoites are released into the body cavity and travel to the mosquito's salivary glands [6, 56]. When a mosquito bites another individual, the human infection cycle starts over again [6, 56] (Figure 2). The life cycle allows the malaria parasite to spread between the mosquito and human, making it difficult to control and eliminate the disease. The clinical signs and symptoms of malaria can vary depending on the stage of the malaria parasite's life cycle and the severity of the infection.

### 4.6. Clinical Signs and Symptoms of Malaria

Initial symptoms of malaria are nonspecific and similar to those of minor systemic viral illnesses (Table 1) making it difficult to diagnose malaria in its early stages, particularly in areas where the disease is common and other illnesses with similar symptoms are prevalent [58].

Classical symptoms are recurrent 6–10 hour attack cycles with three distinct stages, namely, a cold stage (rigors) [59], a hot stage (fever up to 40 degrees Celsius (°C)) [59], accompanied by headaches, vomiting, joint pain, and seizures in young children, and a perspiration stage (sweating, regaining thermal control, and fatigue) [59].

### 4.7. Guidelines for Malaria Diagnosis

Diagnosing malaria can be challenging due to an overlap in signs and symptoms that are also common to other diseases such as viral infections and enteric fever [60]. A delay in the diagnosis and treatment of malaria is the leading cause of malaria deaths [60]. Malaria must be diagnosed promptly to prevent complications from developing. A rapid and effective malaria diagnosis is essential to ease suffering and decrease community transmission [61]. According to WHO guidelines, malaria must be diagnosed with a parasitological test, including light microscopy or immunochromatographic rapid diagnostic tests (RDTs), of which the results must be available [62]. Despite microscopy being historically regarded as the gold standard for diagnosing malaria [63], it is not without its limitations, which include its detection threshold and a lack of infrastructure and skilled personnel in South African laboratories [64]. The use of RDTs somewhat addresses these drawbacks, particularly the logistical difficulties. As a result, both microscopy and RDTs are used in South Africa to diagnose malaria under the National Malaria Diagnostic and Treatment Guidelines [57]. There are several ways to detect and diagnose malaria; however, only a few will be discussed in this review. A clinical assessment (Table 1) is usually performed, followed by a parasitological lab test to confirm the diagnosis.

### 4.8. Microscopic Blood Assay

Conventionally, malaria has been diagnosed through the microscopic examination of Giemsa-stained peripheral blood smears [61, 65]. Parasitic infection is confirmed through thick blood films, whereas species are confirmed through thin blood films [61]. With 100% specificity, microscopic examination remains the gold standard for the diagnosis of malaria [52]. Further advantages of this method are that it is quick, inexpensive, and provides both quantitative (parasite density, with a threshold of 50–500 parasites per microliter (*μ*L)) and qualitative (*Plasmodium* species) data, which allow for the diagnosis of the stage of malaria infection [66, 67].

### 4.9. Rapid Diagnostic Tests

New and cost-effective malaria diagnostic procedures have been identified and developed, namely, the rapid diagnostic test (RDT), which has a specificity of 90% or higher and provides results [68] within 5–20 minutes [69]. The RDT works by detecting a malarial antigen in an individual's blood passing through a membrane that contains specific antimalaria antibodies [70]. This method allows for the identification and detection of plasmodial proteins, specifically histidine-rich protein 2 (HRP-2) and parasite-specific aldolase or parasite-specific lactate dehydrogenase (pLDH) [61]. To perform RDT, a blood specimen acquired from the patient is applied, along with certain reagents, to a sample pad on the test card [71, 72]. The presence of specific bands in the test-card window following a short incubation period indicates whether the patient is infected with *P. falciparum* or one of the other species of human malaria [72]. For *P. falciparum *histidine-rich protein 2 (PfHRP-2), this method has a detection threshold of 2.6–14.6 nanogram (ng) per millilitre (mL) [52].

Of note, deletion of the histidine-rich protein 2 and 3 (HRP-2/3) genes in *P. falciparum* can have significant implications with respect to the diagnosis of malaria. These deletions can lead to the reduced sensitivity of RDT, thus impacting its ability to accurately detect *P. falciparum* infections [73]. For example, false negative results could mean that those individuals that are infected with *P. falciparum* could go undetected and subsequently untreated, leading to the underestimation of malaria cases [74]. It is also important to consider that the prevalence of HRP-2/3 deletions may vary across regions, necessitating tailored diagnostic approaches [73, 75]. In addition, HRP-2/3 deletions could pose a threat to malaria control programmes [76]. These deletions highlight the need to explore alternative diagnostic approaches to increase the reliability of results in regions where these deletions are widespread. Another drawback to RDTs is that the majority of these tests can only detect one malaria species, which is *P. falciparum*, *P. vivax*, human *Plasmodium* species, or a combination of species [75]. Furthermore, this method cannot differentiate between past and present infections and is unable to quantify parasite density [52].

### 4.10. Quantitative Buffy Coat

The quantitative buffy coat (QBC) technique was designed to simplify and enhance the microscopic detection of parasites in peripheral blood and to improve malaria diagnosis [61]. This highly sensitive method is based on microcentrifugation and involves utilising fluorescent dyes, such as acridine orange, to stain parasite deoxyribonucleic acid (DNA) in specialised capillary tubes with a plastic float [77]. The components of the buffy coat segregate according to their densities during centrifugation, generating distinctive bands [78]. Separation and metachromatic labelling of these cells are aided by reagents that cover the QBC test tube [78, 79]. Thereafter, fluorescent microscopy can be used to detect malaria in infected cells and plasma [78].

### 4.11. Immunofluorescence Antibody Testing

Malaria can also be diagnosed using serological methods, which rely on the identification of antibodies against asexual blood-stage malaria parasites [80]. With a specificity of 90–95% and a detection threshold of 100 parasites per *μ*L [52], immunofluorescence antibody (IFA) testing is a time-consuming yet extremely sensitive method for diagnosing malaria [61]. The principle of IFA is that specific antibodies are produced within two weeks of infection with any *Plasmodium* species and remain for 3–6 months after the parasite has been cleared [61]. IFA testing uses a unique antigen produced on a slide, coated, and stored at −30°C before use [61]. In addition, immunoglobulin G (IgG) and immunoglobulin M (IgM) antibodies are quantified in serum samples collected from patients [61]. Despite its usefulness as a species-specific test, this method cannot differentiate between past and present infections [52].

### 4.12. Polymerase Chain Reaction

Molecular diagnostic methods, including the polymerase chain reaction (PCR), have been employed in the molecular diagnosis of malaria, allowing for the precise detection of *Plasmodium* sp. DNA from peripheral blood [81]. It has a specificity of nearly 100% and high sensitivity, requiring only 1–5 parasites per *μ*L for malaria detection [52]. Compared to QBC and RDT methods, the PCR approach was found to be more sensitive and is now widely used to confirm malaria infection, follow-up therapeutic response, and identify drug resistance [61]. A further advantage is that, in cases with very low parasitaemia, PCR may be more sensitive than traditional microscopy and can distinguish between different species [52].

### 4.13. Prevention of Malaria Infection

A combination of pharmacological and nonpharmacological measures is used to prevent malaria in travellers [82]. While pharmacological prophylaxis is extremely vital, nonpharmacological strategies for preventing malaria are just as important; however, they are only meant to supplement existing therapies and should not be used in place of antimalarial drug prophylaxis [37]. It may be beneficial to wear insecticide-treated nets or light-coloured clothing with long sleeves and long trousers [37]. Compared to routinely treated bed nets, long-lasting insecticidal nets (LLINs) and repellents are anticipated to have a longer period of activity [37]. Other forms of protection include avoiding going outside after dusk [83]. Malaria control interventions such as IRS with insecticides and the use of ITNs are known to impact malaria transmission [84]. Insecticides commonly used for LLINs are pyrethroids, and for IRS, they include pyrethroids, carbamates, organophosphates, and DDT [85]. However, emerging vector resistance to these insecticides threatens recent progress in malaria control [85]. Adequate implementation and coverage of these interventions can significantly reduce mosquito populations and the risk of malaria transmission [37]. High-risk patients, including pregnant women, children under the age of five, elderly persons, and immunocompromised persons should avoid exposure to malaria [14]. In addition, people should avoid visiting malaria-endemic countries during the dry season [14].

### 4.14. Treatment of Malaria

Over time, the emergence and spread of drug-resistant malaria parasites, such as *P. falciparum*, have posed significant challenges to malaria control efforts, making malaria drugs less effective in the fight against the disease [86]. This has subsequently led to treatment failures and increased transmission, especially in infected individuals who are not appropriately treated [87, 88]. Addressing drug resistance is crucial to the success of malaria control programmes or interventions, especially in malaria-endemic areas. The steady increase in malarial deaths may be partially attributed to the rise in resistance to sulphadoxine-pyrimethamine, a staple in the treatment and prophylaxis of malaria at the time [89]. The development of resistance has become a major obstacle to the efficient treatment of malaria, particularly malaria caused by *P. falciparum* [90]. Despite advances in understanding the molecular mechanisms of resistance, factors that promote the development and transmission of these resistant parasites remain unclear [91]. Ultimately, resistance led to the introduction of ACT and regional malaria control strategies in parts of Southern Africa [12]. While the signs and symptoms associated with *P. falciparum* infection usually begin between 7 and 21 days subsequent to exposure, longer incubation periods may be observed in patients who failed chemoprophylaxis or received antibiotic therapy during this period [57]. In addition, fewer than half of the travellers who contract malaria pursue pretravel consultation [92]. Individuals who are at risk for malaria include those who do not take the necessary precautions when travelling [83].

As alluded to earlier, *Plasmodium* sp. has a complicated life cycle, and most approved drug treatments target the blood stages by (i) inhibiting haem crystallisation, (ii) acting as antifolates, or (iii) producing free radicals [93]. The inhibitors of haem crystallisation include chloroquine and hydroxychloroquine, as well as amine alcohol derivatives of the same 4-aminoquinoline ring-containing molecule, i.e., lumefantrine, quinine, and mefloquine [93]. All these compounds lead to the accumulation of toxic-free haem in the parasite by preventing its metabolism subsequent to the breakdown of haemoglobin [80, 94]. Antifolates, on the other hand, prevent the production of essential amino acids in the parasite by competing with para-aminobenzoic acid (PABA) for incorporation into folate by inhibiting the dihydropteroate synthase (DHPS; class I antifolates) enzyme or by inhibiting dihydrofolate reductase (DHFR; class II antifolates). The former includes sulphadoxine and dapsone, and the latter includes proguanil and pyrimethamine. However, due to the parasite's ability to scavenge folate from its host, these drugs have limited efficacy when used as monotherapy, and the use of the DHPS inhibitors has largely been negated due to resistance and its propensity for inducing toxicity in individuals [93, 95]. While artemisinin is associated with poor bioavailability, both artemether and artesunate were derived to improve artemisinin's pharmacokinetic properties. Free radicals produced by these compounds induce oxidative stress, which causes damage to proteins and other macromolecular structures within the parasite [80], and ACT therefore remains an important therapeutic option for uncomplicated malaria [8]. Another well-known chemoprophylactic, doxycycline, a tetracycline antibiotic, has been shown to act as a schizonticide during the human blood stage of infection by interfering with protein and pyrimidine biosynthesis. It offers effective chemoprophylaxis against *P. falciparum* even in regions that commonly experience drug resistance, and while being effective in the treatment of acute infection, doxycycline should not be used as monotherapy and is therefore commonly combined with other antimalarials, i.e., quinine or artesunate, for this purpose [93, 96].

The three most prominently prescribed drugs available in South Africa for malaria prophylaxis include doxycycline, atovaquone/proguanil, and mefloquine. While appropriateness is based on the patient profile (Table 2), a combination of atovaquone and proguanil is recommended due to its shorter course of treatment and low incidence of adverse effects. Furthermore, both this combination and doxycycline are available as a pharmacist-advised therapy [99]. Patient compliance and adequate dosing are essential when taking any of these options, as noncompliance results in a significant increase in the failure of chemoprophylaxis [100].

Considering that the majority of malaria cases in South Africa result from *P. falciparum* infections, the treatment guidelines (Tables 3 and 4) are aimed at this species [50, 88].

### 4.15. Antimalarial Drug Resistance

*P. falciparum* has developed resistance to all currently used antimalarial drugs, rendering the use of chloroquine and sulphadoxine-pyrimethamine either alone or in combination ineffective [102]. Resistance to sulphadoxine-pyrimethamine is linked to mutations in the dihydrofolate reductase (DHFR) and dihydropteroate synthase (DHPS) genes, whereas resistance to chloroquine is linked to mutations in the *P. falciparum *chloroquine-resistance transporter (pfcrt) and *P. falciparum* multidrug resistance 1 (pfmdr1) genes [53]. In an attempt to overcome *P. falciparum-*induced resistance, combination treatment has been implemented in several countries including South Africa and is now the preferred approach to treating malaria [103, 104]. The rationale behind combination treatments is to inhibit the spread of parasites resistant to one component of the combination by adding another component with a different mechanism of action. Importantly, the combination will preferably include drugs with similar half-lives so that resistance cannot be acquired when the parasite is exposed to only one drug, with a shorter half-life than the other, for prolonged periods [53]. Advances in uncomplicated *falciparum* malaria treatment include the replacement of chloroquine and sulphadoxine-pyrimethamine with an alternative to chloroquine, namely, ACTs, which are now recommended as first-line treatments, including in pregnancy [102].

Currently used combinations of antimalarial drugs include nonartemisinin combinations, such as quinine and sulphadoxine-pyrimethamine, quinine and doxycycline, sulphadoxine-pyrimethamine and chloroquine, and sulphadoxine-pyrimethamine and amodiaquine, as well as artemisinin-based combinations, such as artemether-lumefantrine, artesunate and amodiaquine, dihydroartemisinin-piperaquine, artesunate and mefloquine, and artesunate and sulphadoxine-pyrimethamine [53]. While *P. ovale*, *P. malariae*, and *P. knowlesi* are still considered to be generally chloroquine-sensitive, patients should be treated with artemether-lumefantrine if infected with *P. vivax* or in regions known to experience chloroquine resistance. If doubt exists about the offending species, standard treatment guidelines for *P. falciparum* infection should be followed. In the case of oral treatment, first doses should be administered under supervision and the patient should be observed for at least an hour due to the common occurrence of vomiting in patients suffering from malaria. Paracetamol is the antipyretic agent of choice due to the increased risk of renal complications with nonsteroidal anti-inflammatory drugs (NSAIDs) in malaria [57]. A recent report by the WHO emphasised the emergence of artemisinin resistance in Africa, which remains a growing concern. However, strategies are currently being put in place to help minimise resistance in countries like South Africa [88].

### 4.16. Novel Antimalarials in Development

As a result of acquired resistance to most of the current malaria treatments and subsequent limited treatment options, the modification of existing treatments as well as the design of novel treatments has emerged (Table 5).

In South Africa, malaria treatment is generally effective, particularly with the use of ACTs [57]. However, the efficacy of treatment can be compromised in cases of drug-resistant strains of the malaria parasite [87]. In terms of affordability, malaria treatment in sub-Saharan Africa poses a significant obstacle [107]. South Africa provides free malaria diagnosis and treatment through its public healthcare system [108]. However, access to healthcare can be limited in certain rural or remote areas, and private healthcare can be expensive for those who cannot afford it [109].

### 4.17. Malaria Vaccine

In October 2021, the WHO recommended the use of the RTS, S/AS01 (RTS, S) malaria vaccine among children living in sub-Saharan Africa and regions with moderate to high *P. falciparum* malaria transmission [110]. This was based on outcomes from a clinical trial conducted in Ghana, Kenya, and Malawi in approximately 900,000 children [110]. The RTS, S vaccine is considered a pre-erythrocytic vaccination because it specifically targets the circumsporozoite protein (PfCSP) on the surface of the sporozoite and *P. falciparum* parasites prior to invading and infecting hepatocytes [111]. The vaccine was created as a virus-like particle (VLP) containing two parts, namely, 18 copies of the central repeat and the C-terminal domain of PfCSP fused to a hepatitis B virus surface antigen [112]. RTS, S/AS01 is the only vaccine that has shown protective efficacy against clinical malaria in a phase III clinical study, albeit protection is very partial, diminishes over time, and might depend on age [112].

### 4.18. Malaria Control and Prevention Programmes

Most children who die from malaria do so because they do not seek treatment promptly. In response, the WHO has suggested implementing Community Case Management of Malaria (CCMm), previously known as home-based management of malaria [113]. This strategy aims to reduce the malaria burden by enhancing early access to malaria-directed healthcare through trained community-based providers like community health extension workers, coordinators, and private vendors. CCMm ensures that effective anti-malarial drugs and referral guidelines are accessible at the community level [113]. The Southern African Malaria Control (SAMC) programme has implemented a systematic and standardised approach to tackle malaria epidemics in the region since 1998 [114]. This comprehensive strategy involves strategic planning, forecasting, prevention, preparedness, and early warning mechanisms to enable a more effective and timely response [114]. Several SAMC countries utilise residual house spraying and insecticide-treated net distribution for preventing epidemics, while residual house spraying is mainly employed as an early response measure to mitigate the impact of outbreaks [114]. Vector control is an essential element of the global malaria control strategy (GMCS) and is widely acknowledged as the most effective approach to preventing malaria transmission [115]. Nevertheless, Ediau et al. reported that inadequate knowledge and negative attitudes toward IRS are prevalent, particularly among individuals living in rural areas with lower education levels [116]. WHO/AFRO (Regional Office for Africa) is promoting the Integrated Disease Surveillance and Response (IDSR) programme, which helps epidemiologists choose and utilise precise indicators, initially on a monthly basis [114]. In districts prone to epidemics, a second step involves gathering weekly malaria morbidity and mortality data to swiftly detect any abnormal rise within two weeks and take prompt action [114]. The programme has great potential to enhance early epidemic detection for malaria and other diseases, and its implementation is advancing effectively across the continent [114].

## 5. Conclusion

Despite South Africa having made significant progress in the control, prevention, and treatment of malaria, this disease continues to pose a serious public health threat. Since 2000, the prevalence of malaria has decreased as a result of significant advancements. However, the issue of drug resistance as well as malaria importation from neighbouring countries still poses a threat. Various chemoprophylactic and treatment approaches are still considered highly effective, albeit subject to patient compliance. With that being said, malaria is preventable and curable if diagnosed and treated early. The Malaria Control Programme in South Africa aims to implement malaria control interventions or measures to help significantly reduce the incidence of malaria in the country. However, despite ongoing efforts to treat malaria and eliminate emerging strains of multidrug resistance, the eradication of malaria remains a challenge in current global efforts to minimise the impact and spread of the disease.

## Figures and Tables

**Table 1:** Diagnostic criteria for suspected cases of malaria (adapted from the algorithm for the management of malaria in South Africa) [57].

Uncomplicated malaria	Severe malaria	
Clinical signs and symptoms		Metabolic and haematological criteria
(i) Mild symptoms (ii) Ambulant (iii) Normal mental function (iv) No repeated vomiting (v) No jaundice (vi) No features of severe malaria	(i) Cerebral malaria (ii) Impaired consciousness (iii) Prostration (iv) Multiple convulsions (>two episodes in 24 hours) (v) Acidotic breathing; respiratory distress (vi) Acute pulmonary oedema; acute respiratory distress syndrome (vii) Circulatory collapse; shock (viii) Anuria (ix) Jaundice (x) Abnormal bleeding	(i) Hypoglycaemia (ii) Metabolic acidosis (iii) Severe normocytic anaemia (iv) Hyperparasitaemia (v) Haemoglobinuria (vi) Hyperlactatemia (vii) Renal impairment

**Table 2:** Available chemoprophylactic options against malaria infection [97, 98].

	Directions for use	Special notes
Doxycycline	100 mg daily starting 2 days prior to entering the endemic area, continued four weeks after return	Do not take along with milk or other dairy or calcium-rich meals; ensure adequate fluid intake and protection against sunburn
		Contraindications: pregnancy and lactation; children <8 years
		Side effects: photosensitivity; gastrointestinal (GIT) disturbances

Atovaquone/proguanil	One tablet (250/100 mg adult) daily starting 2 days prior to entering the endemic area, continued one week after return	Take with milk or fatty foods; repeat dose if vomiting within 1 hour of dosing; caution advised in patients with a history of epilepsy or active treatment with anticoagulants
		Contraindications: children <11 kg; renal impairment
		Side effects: initial gastric intolerance; treatment should be discontinued in the case of a severe rash or mucosal involvement

Mefloquine	One tablet (250 mg) weekly (every 7 days) starting 10 days prior to entering the endemic area, continued four weeks after return	Take after meals with adequate fluid; drug of choice in pregnancy and lactation; avoid activities requiring fine motor coordination
		Contraindications: psychiatric disorders, epilepsy, infants <5 kg; cardiac conduction abnormalities; hepatic impairment
		Side effects: headache, dizziness, vertigo, mood changes, and sleep disturbances; GIT and visual disturbances

**Table 3:** Treatment options for severe malaria infection; doses for adult patients only [50, 88].

	Directions for use	Special notes
Artemether-lumefantrine (Coartem®)	Orally, six doses over three days according to weight; extend to five days in patients >85 kg	Only indicated for cases of uncomplicated malaria; take with milk or fatty foods; for *P. ovale* and *P. vivax* infections, treatment should be followed by primaquine (not registered in SA; available as section 21)
		Warnings/contraindications: caution advised in patients with porphyria or a history of QT abnormalities; coadministration with strong cytochrome P450 3A4 (CYP3A4) inducers may result in treatment failure
		Side effects: sleep disturbances, headaches, dizziness, palpitations, abdominal pain, arthralgia, myalgia

Quinine (Lennon-Quinine Sulphate® 300 mg tablets)	Orally, 10 mg quinine sulphate per kg body weight every 8 hours for 7 to 10 days	For uncomplicated malaria, if artemether-lumefantrine is not available or contraindicated; combined with 100 mg doxycycline or 150 mg clindamycin twice daily after 2 to 3 days (depending on the patient's tolerance thereof)
		Warnings/contraindications: QT abnormalities, glucose-6-phosphate dehydrogenase (G6PD) deficiency, optic neuritis, thrombocytopenia, myasthenia gravis, haemolytic uremic syndrome
		Side effects: cinchonism; headache; nausea; auditory and visual disturbances; unusual sweating; vasodilation

Chloroquine (Nivaquine® 200 mg tablets; Plasmoquine® 200 mg capsules)	Orally, 600 mg stat followed by 300 mg after 6 hours and 300 mg daily on the two following days (1.5 g over three days)	Only for the treatment of uncomplicated infections confirmed not to be *P. falciparum*. Oral primaquine may serve as an alternative (section 21)
		Warnings/contraindications: psoriasis, porphyria, visual disturbances; caution advised in patients with hepatic disease, alcoholism, QT abnormalities, or G6PD deficiency
		Side effects: cinchonism; headache; nausea; visual disturbances

Artesunate (Garsun 60 mg injectable)	2.4 mg/kg (3 mg/kg if the patient <20 kg) intravenous (IV) at 0, 12, and 24 hours followed by once daily until the patient can tolerate oral treatment	Artesunate is considered safe and tolerable and rarely leads to severe adverse events and fewer neurological defects
		Warnings/contraindications: possible delayed haemolysis, strong UGT inducers may reduce efficacy, foetal toxicity demonstrated in animals but not confirmed teratogenicity in humans
		Side effects: GIT disturbances, dizziness, haematological disorders very rare

Quinine (IV) (Quinine Dihydrochloride-Fresenius® 300 mg/mL)	Loading: 20 mg/kg quinine dihydrochloride diluted 10 mL/kg bodyweight 5% dextrose over 5 hours Maintenance: started 8 hours after initiating loading dose; 10 mg/kg in dextrose over 4 hours; administered 8-hourly until the patient can tolerate oral treatment	Reserved for severe malaria. Never to be administered as bolus injection; intramuscular route may be used as an alternative if required (refer to guidelines); reduce dose to 10 mg/kg 12-hourly on the third day if parenteral treatment is required for longer than 48 hours
		Warnings/contraindications: narrow therapeutic window; QT abnormalities, G6PD deficiency, optic neuritis, thrombocytopenia, myasthenia gravis, haemolytic uremic syndrome
		Side effects: cinchonism; headache; nausea; auditory and visual disturbances; unusual sweating; vasodilation; hypoglycaemia

**Table 4:** Treatment options for uncomplicated malaria infection in pregnancy [50, 101].

Malaria species	Trimester	Treatment	Directions for use	Special notes
*P. falciparum*	First	Quinine and clindamycin	7-day treatment	Quinine can be used alone and can be used if clindamycin is not available. In cases of failure or unavailability, artemisinin-based combination therapy or oral artesunate with clindamycin can be used for 7 days, as artemisinin derivatives have not shown risk for major congenital defects
Non-*falciparum* malaria	First	Chloroquine	As described in Table 3	Can be replaced with quinine for chloroquine-resistant infections
*P. falciparum*	Second and third	ACTs as the first-line treatment, the same as for nonpregnant adults	Any ACTs can be used in pregnancy	The mean birthweight was significantly higher in patients using ACTs, as ACTs might clear parasites (including placental parasites) more efficiently than other treatments

**Table 5:** Novel antimalarials in development [102, 105, 106].

Drug class	Drug names	Mechanism of action	Notes
Malaria parasite protease inhibitors	(i) Cysteine and serine protease inhibitor (leupeptin) (ii) Cysteine protease inhibitor (E-64, epoxomicin, lactacystin, MG132, WEHI-842, WEHI-916, chymostatin) (iii) Aspartic protease inhibitor (pepstatin) (iv) Serine protease inhibitor (LK3)	Drugs in this class disrupt malarial proteases, which inhibit haemoglobin degradation by intraerythrocytic trophozoites and the parasite development in the erythrocyte stages	A cysteine protease inhibitor (E-64) and an aspartic protease inhibitor (pepstatin) previously demonstrated synergistic inhibition towards *P. falciparum* development. In addition, E-64 blocked globin hydrolysis. Previous studies have indicated that several cysteine protease inhibitors inhibited both *P. falciparum* growth and haemoglobin degradation

Phosphatidylinositol 4-kinase (PfPI4K) inhibitor	(i) UCT943 (ii) Imidazopyrazines (KAF156) (iii) Aminopyridine (iv) Compound 1294	Inhibition of phosphoinositide lipid kinases (PIKs) inhibits the activation of lipids by preventing lipid phosphorylation and subsequently inhibits proliferation, survival, trafficking, and intracellular signalling	PI(4)K inhibitors block the intracellular development of multiple *Plasmodium* species at each stage of infection in the host KAF156 is currently in phase II clinical trials Compound 1294 previously inhibited the transmission of parasites from mosquitoes to humans

Transporter inhibitors	(i) Anion transporter inhibitors (phlorizin, dantrolene, furosemide, and niflumate) (ii) Inhibitors of choline influx into parasite-infected erythrocytes (glibenclamide, meglitinide, and tolbutamide)	Transporter inhibitors inhibit transporters, such as the plasmodial surface anion channel (PSAC) and the parasitophorous vacuolar membrane (PVM), which are essential for the entrance of metabolites, electrolytes, and nutrients into the parasite	Of these transporters, PSAC is the most promising target due to its critical role in several types of nutrient acquisition into the intracellular parasite as well as in maintaining a low Na^+^ and K^+^ permeability ratio in parasites

*Plasmodium* sugar transporter inhibitor	(i) Long-chain O‐3‐hexose derivative (compound 3361)	Inhibits the uptake of glucose by *P. Falciparum* hexose transporter (PFHT), as *P. falciparum* depends on glycolysis (the uptake of glucose) for replication	Compound 3361 previously inhibited the uptake of glucose and fructose by PFHT in *P. vivax,* induced death in *P. falciparum*, and reduced multiplication of *P. berghei* in a mouse model

Parasite's lactate transporter inhibitor	(i) MMV007839 (ii) MMV000972	Inhibition of the lactate H^+^ symport transport system inhibits lactate export and glucose uptake in the parasite	Compounds, such as MMV007839 and MMV000972, previously induced death in *P. falciparum*

P-type Na^+^ ATPase inhibitor (PfATP4)	(i) Cipargamin (ii) (+)-SJ733 (iii) KAE609	Inhibition of P-type ATPase transporter (PfATP4) inhibits the parasite's primary Na^+^-efflux pump, leading to increased cytoplasmic Na^+^ levels and subsequent death	KAE609 and cipargamin are currently in phase II clinical trials and were previously shown to display accelerated parasite clearance compared to artemisinins

V-type H^+^-ATPase inhibitor	(i) MMV253	V-type H^+^-ATPase inhibitors inhibit H^+^ efflux in parasites	MMV253 previously inhibited the V-type H^+^ ATPase through mutant selection and whole-genome sequencing

Aquaporin-3 inhibitor	(i) Auphen	Aquaporin-3 inhibitors inhibit the entry of glycerol into *P. berghei* and contributes to the replication of the parasite during the asexual intraerythrocytic stages	Aquaporin-3 inhibitor, Auphen, previously inhibited *P. berghei* in hepatocytes and *P. falciparum* in erythrocytes

Choline transport inhibitor	(i) Albitiazolium (ii) G25 (iii) T3	Choline transport inhibitors inhibit choline influx into the parasite, which is essential for the production of phosphatidylcholine (an important component of parasite cell membranes)	Albitiazolium is currently in phase II clinical trials G25 previously exhibited potent and highly selective cytotoxicity against *P. falciparum* and *P. vivax in vitro*

Dihydroorotate dehydrogenase inhibitor	(i) DSM190 (ii) DSM265 (iii) P218	Dihydroorotate dehydrogenase inhibitors inhibit *de novo* pyrimidine synthesis in parasites, leading to death	DSM265 is currently in phase II clinical trials

Isoprenoid biosynthesis inhibitor	(i) Fosmidomycin (ii) MMV019313 (iii) MMV008138	Isoprenoid biosynthesis inhibitors inhibit 1-deoxy-D-xylulose-5-phosphate (DOXP) reductoisomerase in pathways specific to *P. falciparum* for asexual replication	MMV019313 previously displayed selective cytotoxicity towards parasites

*P. falciparum* translational elongation factor 2 inhibitor	(i) Sordarin (ii) M5717	*P. falciparum* elongation factor 2 inhibitors inhibit a ribosome component responsible for catalysing the GTP-dependent translocation of the ribosome along messenger RNA and thus inhibit protein synthesis in eukaryotes	M5717 is currently in phase I clinical trials

## Data Availability

No underlying data were collected or produced in this study.
